# Gasdermin D protects against noninfectious liver injury by regulating apoptosis and necroptosis

**DOI:** 10.1038/s41419-019-1719-6

**Published:** 2019-06-17

**Authors:** Chenxuan Yang, Ping Sun, Meihong Deng, Patricia Loughran, Wenbo Li, Zhongjie Yi, Shilai Li, Xianghong Zhang, Jie Fan, Timothy R. Billiar, Melanie J Scott

**Affiliations:** 1 Department of Surgery, University of Pittsburgh, Pittsburgh, PA USA; 2 Tsinghua University School of Medicine, Beijing, China; 3 Research and Development, Veterans Affairs Pittsburgh Healthcare System, Pittsburgh, PA USA; 4 Pittsburgh Liver Research Center, University of Pittsburgh, Pittsburgh, PA USA

**Keywords:** Cell death and immune response, Innate immunity

## Abstract

Gasdermin D (GsdmD) was recently identified as the executioner of pyroptotic inflammatory cell death, and is a substrate for caspases-1 and 11. GsdmD is detrimental in lethal endotoxemia but protective in bacterial sepsis. However, little is known about its role during noninfectious/sterile injuries. In this study, we examined the contribution of GsdmD using WT and GsdmD^−/−^ mice in two models of noninfectious liver injury: hemorrhagic shock with resuscitation (HS/R) and acetaminophen (APAP) overdose. GsdmD^−/−^ mice had significantly increased liver damage at 6 h after HS/R or APAP vs WT, shown by significantly elevated ALT level and extended areas of cell death in liver. Caspase-8, a mediator of multiple cell death pathways, was highly elevated in GsdmD^−/−^ mice after injury. Significantly increased cleavage of caspase-8 and subsequent high levels of apoptosis were found in livers of GsdmD^−/−^ mice after HS/R, a relatively mild ROS-induced liver injury. However, during more severe APAP-mediated ROS-induced liver injury, caspase-8 cleavage in GsdmD^−/−^ liver was inhibited compared with WT, resulting in accumulation of pro-caspase-8 and increased levels of necroptosis. Our findings indicate a novel hepatoprotective role for GsdmD in noninfectious inflammation models via regulation of caspase-8 expression and downstream cell death pathways. The effects of GsdmD protection are likely injury specific and may also depend on injury severity and levels of ROS produced. These data suggest modulation of GsdmD/caspase-8 may be a novel therapeutic option in ROS-mediated liver injury.

## Introduction

Gasdermins are a conserved family of proteins^1^ mainly expressed in epithelial tissues. Six gasdermin proteins have been characterized, which mainly function to regulate cell proliferation and differentiation^2^. Gasdermin D (GsdmD) has recently been revealed as a key regulator of inflammation and inflammatory cell death, pyroptosis^3^. During inflammatory conditions, GsdmD is cleaved by inflammatory caspases, caspase-1 and caspase-11 (4/5 in humans), allowing its N-terminus fragments to oligomerize and insert in cell membranes to form pores, resulting in inflammatory pyroptotic cell death^4^. GsdmD N-terminus also has an affinity for cardiolipin-rich membranes (e.g., on bacteria), and so can also be antimicrobial through pore formation and direct lysis of bacteria^5^. This is an important beneficial and protective mechanism of GsdmD in intracellular bacterial clearance (e.g., in *Listeria monocytogenes* infection)^4^. However, in LPS-induced inflammation GsdmD was not protective, with GsdmD-deficient (GsdmD^−/−^) mice showing improved survival in lethal endotoxemia with reduced inflammatory mediator release from pyroptotic immune cells^6^. The contribution of GsdmD in noninfectious/sterile injuries remains unclear and has not been well studied to date.

Acetaminophen (APAP) is one of the most widely used analgesics, and APAP overdose is the leading cause of acute liver failure in resource-rich countries^7^. APAP overdose induces severe ROS-induced liver damage through metabolic depletion of hepatocyte glutathione, an important antioxidant required for hepatocyte redox homeostasis^8^. Hemorrhage is a common complication in traumatic injuries, and can result in hemorrhagic shock characterized by hypoperfusion and hypoxia in multiple organs, including the liver^9^. Hemorrhage is usually treated with fluid resuscitation to increase blood pressure and cellular perfusion^10^. However, resuscitation also increases damaging ROS production, leading to secondary organ injury^11^. In this study, we used these two liver injury models in mice, APAP overdose and hemorrhagic shock with resuscitation (HS/R), which differ in the severity of ROS-induced liver damage, to assess the role of GsdmD.

Various studies have reported the role of inflammasomes in APAP overdose and HS/R. Our group showed previously that during HS/R caspase-1 activation is hepatoprotective through induction of mitophagy and removal of ROS-producing mitochondria^9^. In this model, AIM2 inflammasome in hepatocytes, and not the more extensively characterized NLRP3 inflammasome, was the main activator of caspase-1^12^. The role of inflammasomes during APAP overdose appears more complex^13^. Early publications suggested hepatocyte cell death after APAP was exacerbated by NLRP3 inflammasome and TLR9 signaling^14^. Since then, however, other groups have suggested NLRP3 and IL1β are not required for secondary inflammation following APAP-induced hepatocyte cell death^13,15,16^. Multiple types of cell death occur in APAP injury, including initial necrosis, followed by pyroptosis, apoptosis and necroptosis^17^. However, none has focused on the role of the inflammasome downstream executor GsdmD during HS/R or APAP overdose.

In contrast to its detrimental role in lethal endotoxemia, we show here that GsdmD^−/−^ mice had significantly increased liver damage after both HS/R and APAP overdose, suggesting a protective effect of GsdmD. Furthermore, we show that GsdmD-mediated protection is through its regulation of both apoptosis and necroptosis pathways via regulation of caspase-8 expression and activation, which depends on the severity of injury and ROS production. Our data shed new light on the complexity of distinct yet interrelated programmed cell death pathways, and suggest modulation of GsdmD activation could be a potential therapeutic target during noninfectious liver injury.

## Methods and materials

### Animals, hemorrhagic shock, and APAP-induced hepatotoxicity

Male C57BL/6 (WT) mice were purchased from Jackson Laboratory. GsdmD^–/–^ mice were bred in our facility. Mice aged 8–12 weeks, weighing 21–30 g, were used in our experiments. WT mice were used as controls for genetic knockout mice bred in our facility and were given 2 weeks’ acclimation to the breeding facility prior to experimentation. All experimental protocols were approved by the Institutional Animal Use and Care Committee of the University of Pittsburgh. Experimental procedures were carried out in accordance with all regulations regarding the care and use of experimental animals (National Institutes of Health). HS/R surgery was performed as previously described^9^. Briefly, mice were bled via femoral artery cannulation to a mean arterial pressure of 25 mmHg for 1.5 h, followed by resuscitation with 3× shed blood volume of Ringer’s lactated solution. Mice were sacrificed at 4.5 or 24 h after resuscitation with collection of blood and liver. Control mice were sacrificed without any procedures performed to obtain physiological baseline levels. For APAP-induced hepatotoxicity, mice were fasted for 14–16 h with free access to water. APAP solution was prepared fresh for each experiment in 0.9% saline and administered in a single intraperitoneal injection (400 mg/kg). Controls received solvent in 0.9% saline. Mice were sacrificed at 6 or 12 h after treatment with collection of blood and liver.

### Reagents

APAP and hydrogen peroxide were from Sigma (St. Louis, MO, USA). Z-VAD(OMe)-FMK (Z-VAD) and *N*-Acetyl-L-Carnosine (NAC) was from Cayman Chemical (Ann Arbor, MI, USA). Hi-Perfect transfection reagent was from Qiagen (Germanton, MD, USA). GsdmD siRNA and SCR control siRNA were from Origene (Rockville, MD, USA). Primary antibodies used were anti-cleaved caspase-3, anti-RIP, anti-phosphorylated RIP, anti-MLKL, anti-phosphorylated MLKL, anti-caspase-8, anti-cleaved caspase-8 from Cell Signaling Technologies (Danvers, MA, USA), and anti-gasdermin D from Santa Cruz Biotechnology (Dallas, TX, USA).

### Western blotting analysis

For in vitro experiments, hepatocytes were washed with cold phosphate-buffered saline (PBS) at the endpoint of the experiments, collected in lysis buffer (Cell Signaling Technology) and centrifuged at 16,000 × *g* for 10 min; the supernatant was collected for Western blotting. For in vivo experiments, frozen liver (median lobe) was homogenized in lysis buffer and centrifuged at 16,000 × *g* for 10 min, and supernatant was collected. Protein concentrations from the supernatants were determined with the BCA (bicinchoninic acid) protein assay kit (Thermo Fisher Scientific, Waltham, MA, USA). SDS loading buffer was then added to the samples. Denatured protein samples were analyzed by 10% or 15% SDS–polyacrylamide gel electrophoresis and then transferred onto a polyvinylidene difluoride membrane at 250 mA for 2 h. The membrane was blocked in 5% milk (Bio-Rad, Hercules, CA, USA) for 1 h and then incubated overnight with primary antibody in 1% milk. Membranes were washed three times in TRIS-buffered saline containing Tween (TBS-T) for 10 min, incubated with horseradish peroxidase-conjugated secondary antibody along with fluorescent housekeeping gene antibody (Bio-Rad) for 1 h, and then washed three times in for 10 min in TBS-T, before being developed for chemiluminescence (Bio-Rad). Western images were quantified by densitometry using ImageJ software (National Institutes of Health).

### Hepatocyte isolation and cell culture

Hepatocytes were isolated from mice by an in situ collagenase (type VI; Sigma) perfusion technique, modified as previously described^11,18^. Hepatocyte purity exceeded 99% as measured by flow cytometry. The cell viability is typically over 95% by trypan blue exclusion. Hepatocytes (150,000 cells/ml) were plated on gelatin-coated culture plates in Williams-E medium with 10% calf serum, 15 mM Hepes, 1 µM insulin, 2 mM L-glutamine, 100 U/mL penicillin, and 100 U/mL streptomycin. Hepatocytes were allowed to attach overnight, then the medium was replaced with fresh medium before experimental treatment. Hypoxia with reoxygenation (H/R) treatment was performed by culturing hepatocytes under hypoxia (1% oxygen) in a hypoxia chamber. Cells are then reoxygenated under normoxic conditions in the standard cell culture incubator.

### LDH assay

Lytic cell death was measured using an LDH-Cytotoxicity Assay Kit (Abcam) according to the manufacturer’s instructions and analyzed by a spectrophotometer (Biotech).

### ALT and cytokine abundance assessment

ALT level was measured with Heska Lab Systems. Cytokine abundance was analyzed by ELISA specific for interleukin (IL)-6 (R&D Systems Inc.) and high mobility group box 1 (HMGB1) (MBL Inc.) in the plasma samples according to the manufacturer’s instructions.

### Histological analysis

Livers from mice were removed after perfusion with cold PBS and 2% paraformaldehyde. These same samples were further fixed in 2% paraformaldehyde for an additional 2 h and then switched to 30% sucrose in distilled water solution for 24 h^19^. Samples were sectioned to six µm and stained with hematoxylin and eosin (H&E). Imaging was performed using the large-area functionality of a Zeiss Axio Imager 2 (×20, Zeiss, Oberkochen, Germany) using the operational software TissueFaxs (TissueGnostics, Vienna, Austria). A postproduction two-fold digital zoom was generated in NIH Image J shareware.

### Immunofluorescence

Samples were sectioned at six µm and permeabilized with 0.1% Triton X-100 for 20 min, followed by five washes with PBS + 0.5% BSA (PBB). Tissue sections were analyzed using the In Situ Cell Death Detection Kit-TMR red (Cat no. 12156792910, Roche, Germany) or anti-4-HNE antibody following the manufacturer’s instructions. Briefly, tissue was incubated in reaction mixture containing TdT and Tetramethylrhodamine (TMR)-conjugated nucleotides for 1 h at 37 °C. Tissue sections were then washed twice in PBS for three times and the nuclei were stained with a 15 s incubation with 1 mg/ml Hoechst H 33342 (Sigma-Aldrich, Cat no. B-2883, St. Louis, MO, USA). Finally, after a PBS rinse the sections were coverslipped using Aquamount mounting media. For controls, sections were treated excluding the primary antibody (“primary delete”), and the samples were imaged with microscope settings to minimize sample autofluorescences using the primary delete processed sample. Imaging conditions were maintained at identical settings with original gating performed using the negative control. Imaging was performed using a Nikon A1 confocal microscope (×20 with a 2 digital zoom, purchased with 1S10OD019973-01 awarded to Dr. Simon C. Watkins). Quantification was performed using NIS Elements (Nikon, Melville, NY, USA). The number of TMR-positive cells that colocalized with Hoechst positive nuclei were normalized by the number of total nuclei. The final figures were imported as Tiff format and assembled in Adobe Photoshop.

### Statistical analysis

Results are displayed as mean ± SD from at least three independent experiments. Data were analyzed by GraphPad Prism (GraphPad software). Otherwise indicated, a two-tailed Student *t*-test was used to calculate the statistical significance of two experimental groups. *P* < 0.05 was considered significant.

## Results

### GsdmD^−/−^ mice have increased liver damage after non-infectious liver injury

**Fig. 1:**
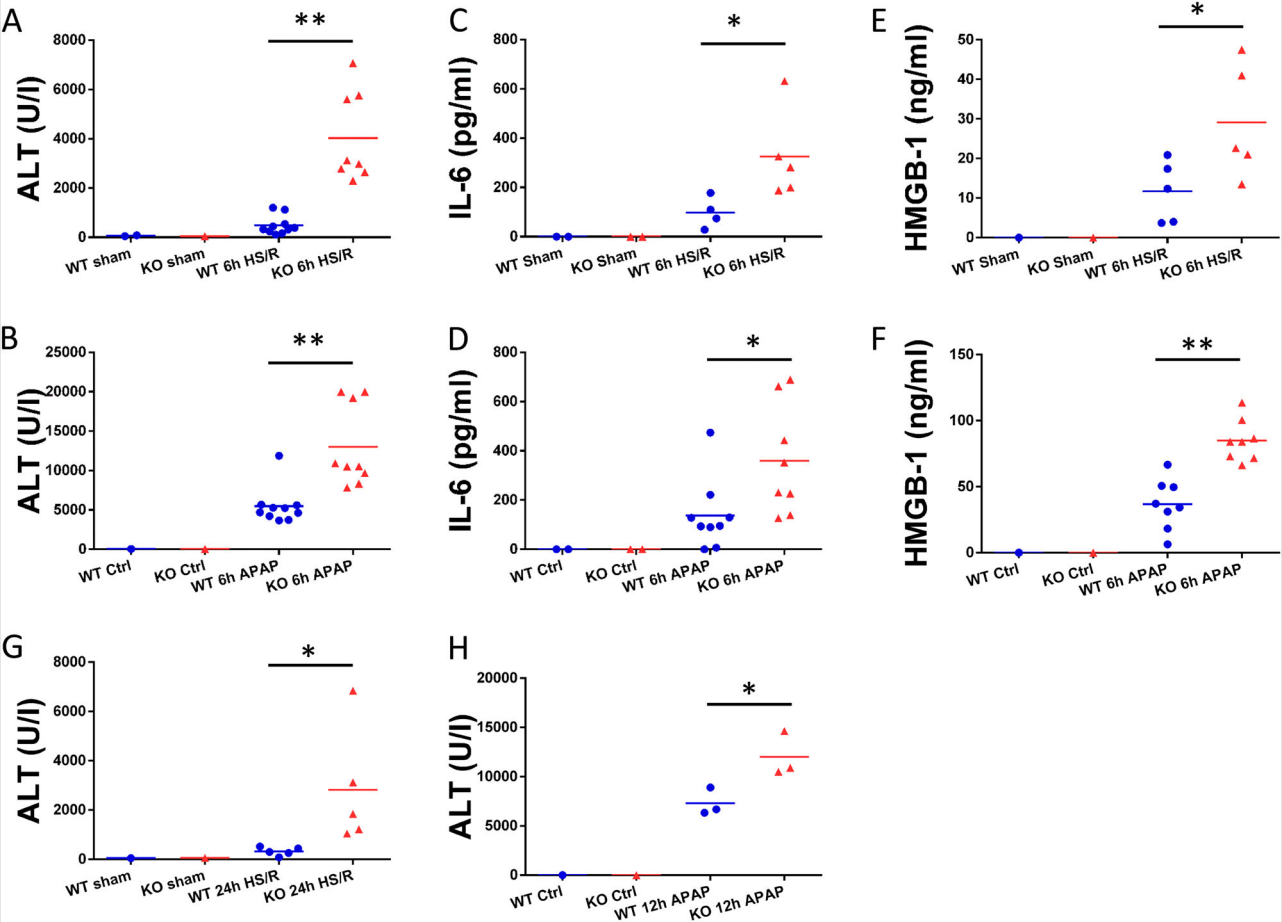
Liver damage and systemic inflammation in WT and GsdmD^−/−^ (KO) mice after 6 h hemorrhagic shock with resuscitation (HS/R) or acetaminophen overdose (APAP). **a**, **b** Circulating ALT level; **c**, **d** circulating IL-6 level; and **e**, **f** circulating HMGB1 level in WT and GsdmD^−/−^ mice at 6 h after HS/R or APAP. **g**, **h** Circulating ALT level in WT and GsdmD^−/−^ mice at 24 h after HS/R or 12 h after APAP. Each symbol in the graphs represents an individual mouse. *N* = 3–10 per experimental group. Horizontal bar shows mean. *p < 0.05 between indicated groups; **p < 0.001 between the indicated groups

To determine the role of GsdmD in noninfectious liver injuries, we used two established clinically -relevant noninfectious liver injury models, APAP overdose and HS/R, and assessed liver damage and systemic inflammation in WT (C57BL/6) and GsdmD^−/−^ mice. Surprisingly, as opposed to the protective phenotype of GsdmD^−/−^ mice in lethal endotoxemia, we found significantly increased circulating ALT levels at 6 h after HS/R (Fig. 1a), suggesting increased liver injury in GsdmD^−/−^ compared with WT. Circulating IL-6, indicative of systemic inflammation, was also increased in plasma 6 h after HS/R (Fig. 1b), and circulating HMGB1 level was also significantly higher (Fig. 1c). Consistent with HS/R, circulating ALT after APAP overdose was also significantly increased in GsdmD^−/−^ mice compared with WT (Fig. 1d). The severity of liver injury after APAP overdose was significantly higher than HS/R, with higher overall ALT levels. Both plasma IL-6 (Fig. 1e) and HMGB1 (Fig. 1f) were also increased in GsdmD^−/−^ mice after 6 h of APAP overdose.

To understand whether the phenotype is preserved at later time points, we harvested tissue and plasma at 24 h after HS/R, or at 12 h after APAP overdose. The phenotype is mostly intact at these later time points with elevated circulating ALT after HS/R (Fig. 1g) and APAP overdose (Fig. 1h) in GsdmD^−/−^ mice. Together these data suggest a protective role for GsdmD in noninfectious injury. This protective effect is conserved between models with different severities of liver injury and at longer time points after injury.

### GsdmD deficiency increases hepatocyte death in noninfectious liver injury

**Fig. 2:**
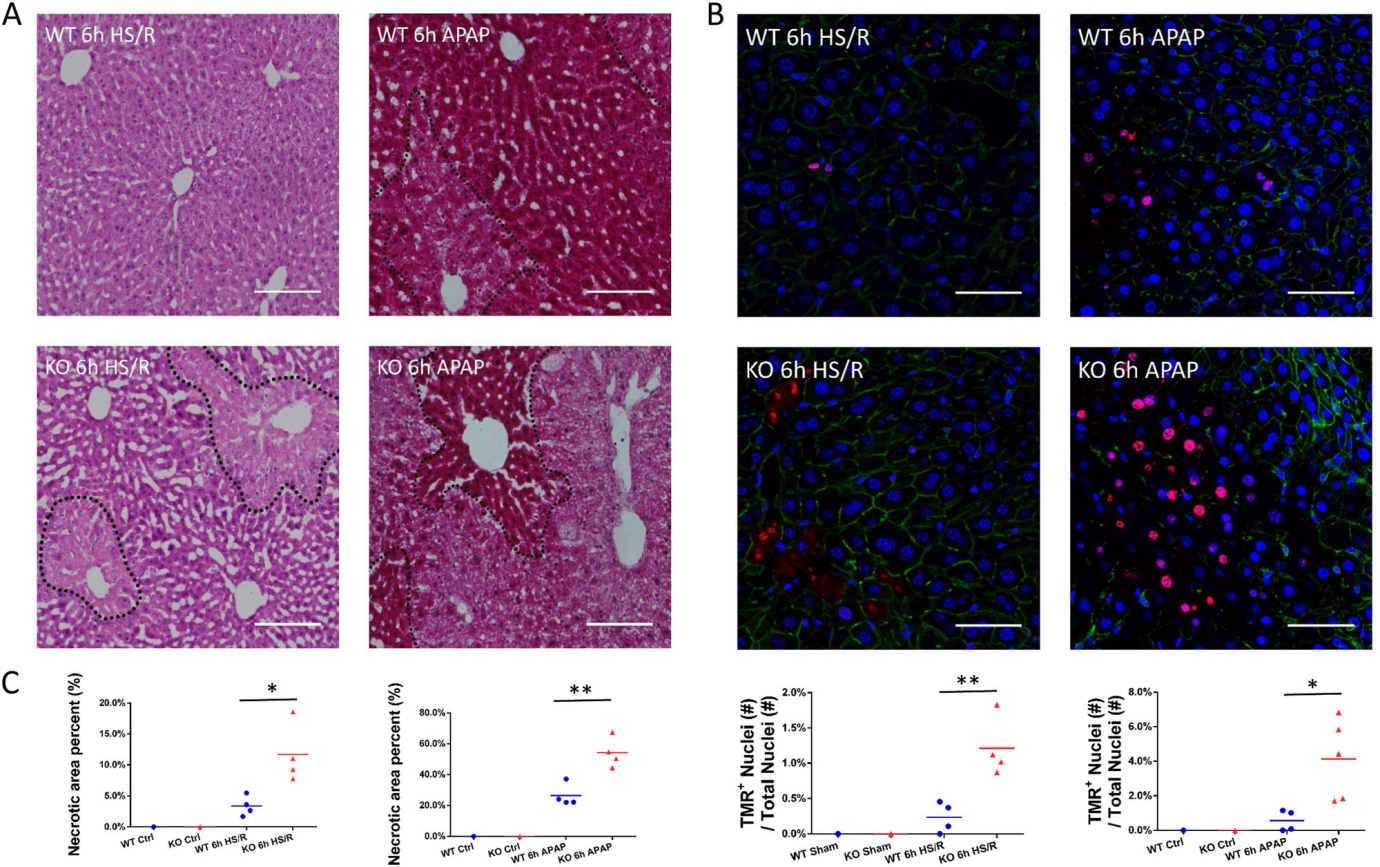
**a** Liver histology (H&E) in WT and GsdmD^−/−^ (KO) liver at 6 h after hemorrhagic shock with resuscitation (HS/R) or acetaminophen overdose (APAP). Lower graphs show quantification of necrotic area. Each point represents mean values for an individual mouse across at least ten fields of view. Horizontal bar represents overall mean value. **b** Liver immunofluorescent TMR staining in WT and GsdmD^−/−^ liver at 6 h after HS/R or APAP. Red = TMR-positive cells; blue = nuclei; green = actin. **c** Lower graphs show quantification of TMR-positive nuclei as a percentage of total nuclei. Each point represents mean values for an individual mouse across at least ten fields of view. Horizontal bar represents overall mean value. Scale bar, 40 μm. 20× Magnification. Dotted lines in H&E images outline necrotic areas. All images are representative of each group. *N* = 4/group. *p < 0.05 between indicated groups; **p < 0.001 between the indicated groups

After 6 h HS/R, H&E staining of liver sections showed oncosis and minor necrosis in WT mice, but GsdmD^−/−^ mice had much larger areas of pericentral necrosis (Fig. 2a). Similarly, after 6 h APAP overdose, WT livers showed a moderate level of pericentral necrosis, with extended necrotic areas in GsdmD^−/−^ mice (Fig. 2a). Control and sham groups of both WT and GsdmD^−/−^ showed normal liver morphology with no necrosis.

To determine hepatocyte cell death in liver, we used TMR staining (similar to TUNEL) in the liver sections after HS/R or APAP overdose. Consistent with the previous data, TMR-positive cells were significantly increased in GsdmD^−/−^ compared with WT mice at 6 h after both HS/R or APAP overdose (Fig. 2b), indicating more DNA damage and hepatocyte cell death in GsdmD^−/−^ mice, consistent with increased circulating HMGB1. Again, WT and GsdmD^−/−^ mice showed similar differences at later time points after HS/R and APAP overdose (Supplemental Fig. 1a–c).

### GsdmD deficiency contributes to differential regulation of apoptosis and necroptosis

GsdmD has been shown as the executioner of pyroptosis^5^. However, recent reports associated signaling pathways linking pyroptosis with other forms of programmed cell death, including apoptosis and necroptosis^20,21^. As we observed more hepatocyte cell death during noninfectious injury in GsdmD^−/−^ mice, we asked what cell death pathways contributed to this and whether defective pyroptosis will result in compensation of other forms of cell death.

**Fig. 3:**
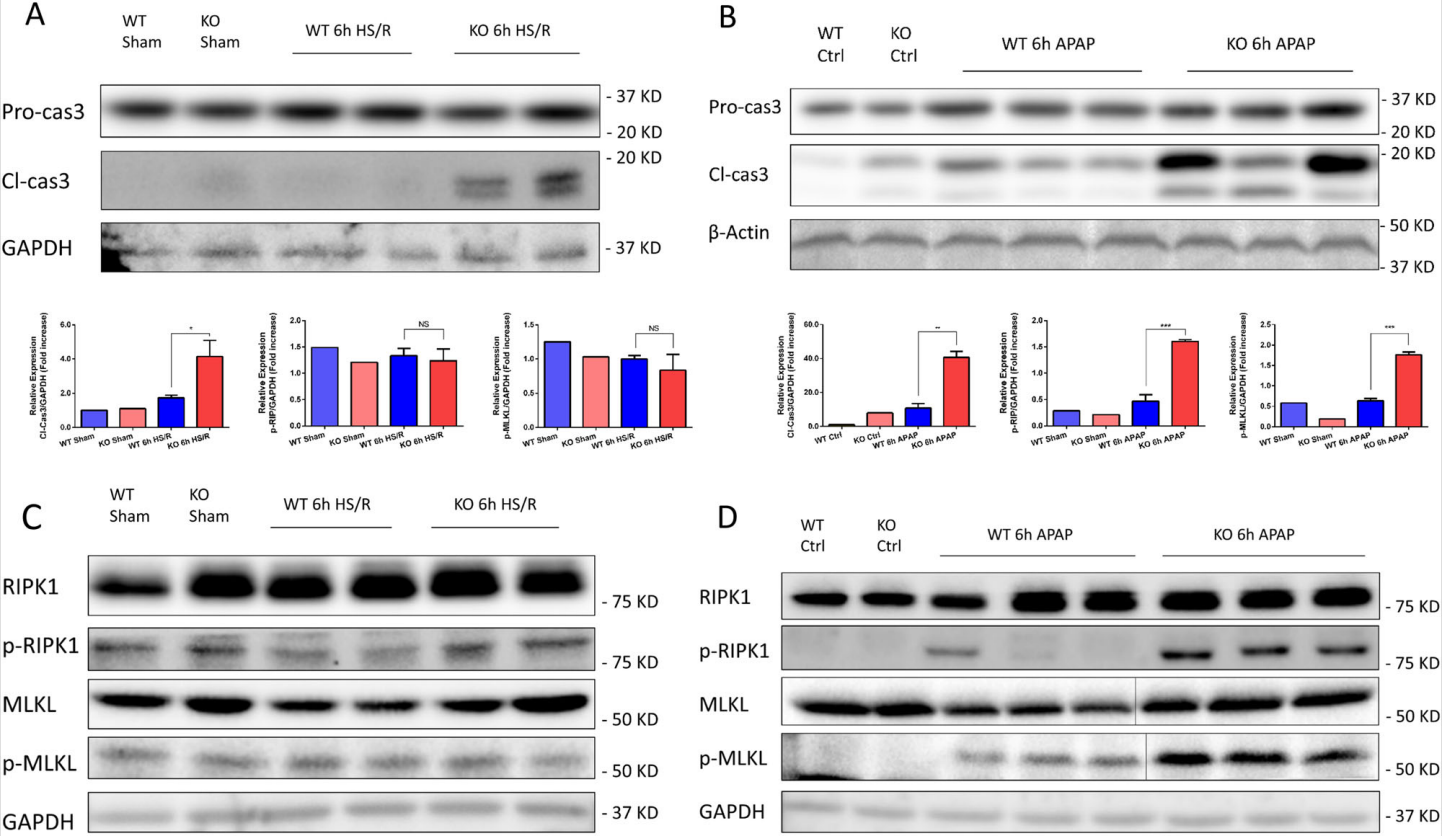
Western blots of whole-cell lysates from the liver of WT and GsdmD^−/−^ (KO) mice at 6 h after hemorrhagic shock with resuscitation (HS/R) or sham surgery (Sham), or 6 h after acetaminophen overdose (APAP) or controls. **a**, **b** Full-length (pro-)caspase-3 (Pro-cas3) and cleaved/active caspase-3 (Cl-cas3) levels. **c**, **d** Total RIPK1 (RIPK1) and MLKL (MLKL), and phosphorylated/active RIPK1 (p-RIPK1) and MLKL (p-MLKL) as markers of necroptosis. GAPDH or β-actin was used as loading controls. Images representative of results from at least three repeats. Each lane contains liver from one individual mouse. *N* = 4–5/group. Quantitation of band density was performed across at least three separate blots and mean ± SEM is shown in the graphs. NS = not significant. *p < 0.05, **p < 0.01, ***p < 0.001 compared with the indicated groups

Apoptosis plays crucial roles in both physiological and pathological states^22^. Caspase-3 is the major executioner of apoptotic cell death^23^, so we measured the activation/cleavage of caspase-3 in WT and GsdmD^−/−^ liver after HS/R or APAP overdose. Surprisingly, in both models GsdmD^−/−^ mice had increased liver apoptosis compared with WT, with increased cleaved caspase-3 by immunoblot (Fig. 3a, b). Similarly, increased cleaved caspase-3 was also observed in cultured isolated hepatocytes from WT and GsdmD^−/−^ mice in vitro after treatment with hypoxia/reoxygenation or APAP (Supplemental Fig. 2, b). These data suggest that much of the cell death signaling observed in liver in vivo is likely due to activation of these pathways in the main cell type in the liver, hepatocytes. These data suggest compensatory upregulation of apoptosis in hepatocytes/liver upon cellular stress in the absence of GsdmD.

Necroptosis or ‘programmed necrosis’, mediated by the necroptosome containing RIP and its target protein MLKL, leads to highly inflammatory cell death^24^. To determine activation of the necroptosis pathway, we used immunoblot to measure levels of activated/phosphorylated RIPK1 and MLKL. While the level of phosphorylated RIPK1 and MLKL remained at baseline levels in both WT and GsdmD^−/−^ mice after 6 h of HS/R (Fig. 3c), we found significant increases of both phospho-RIPK1 and -MLKL in GsdmD^−/−^ mice after 6 h of APAP overdose, compared with WT (Fig. 3d). Results were similar at later time points (Supplemental Fig. 1f, g).

These data suggest GsdmD protects hepatocytes from noninfectious injuries by inhibiting alternative cell death pathways including apoptosis and necroptosis, both of which are upregulated with GsdmD deficiency. However, protection is dependent on severity of injury, with necroptosis induced only in more severe injury.

### Caspase-8 is elevated in the absence of GsdmD and regulates apoptosis and necroptosis

**Fig. 4:**
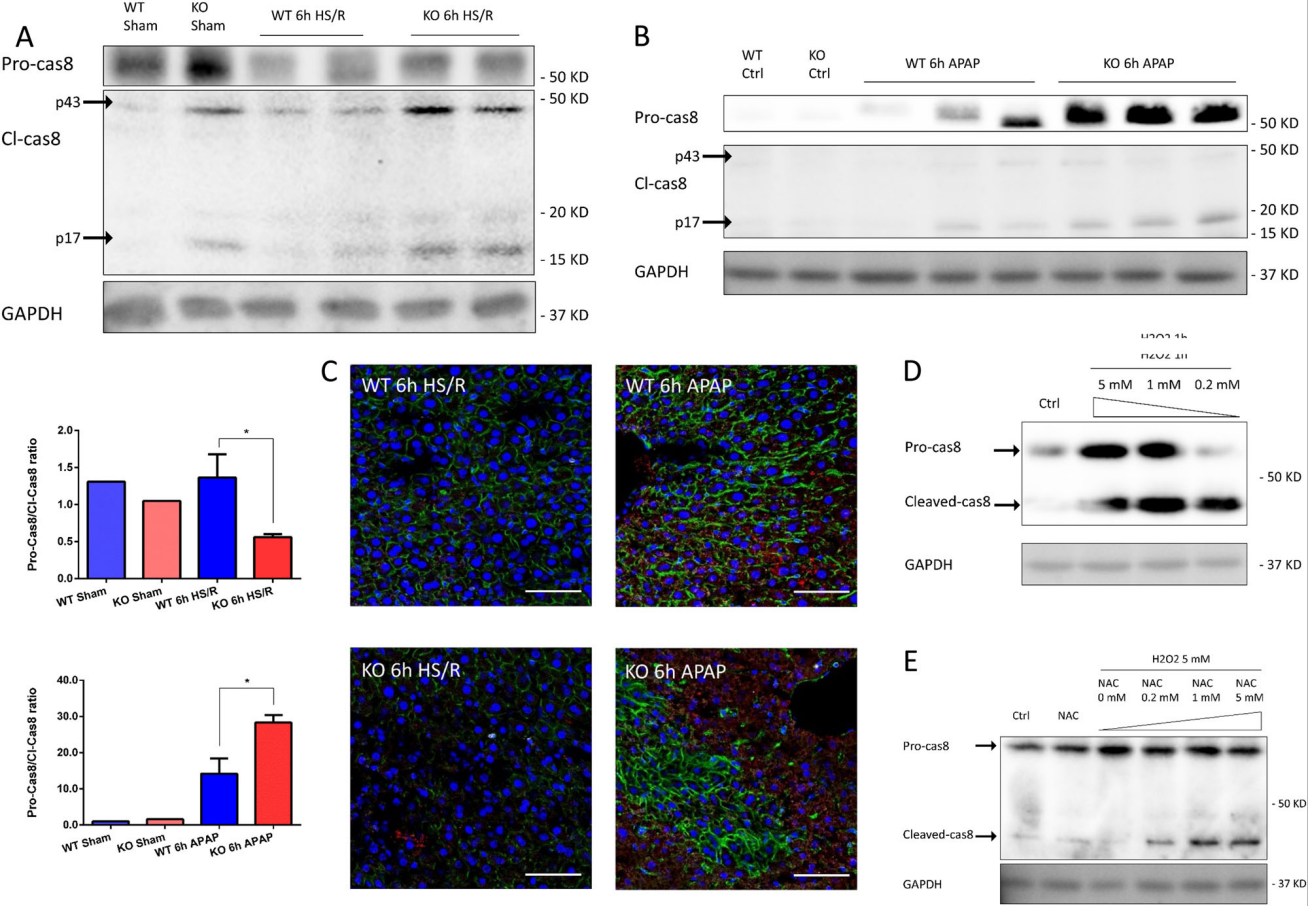
Western blots of full-length (pro-)caspase-8 (Pro-cas8) and cleaved caspase-8 (Cl-cas8) in whole-cell lysates from liver of WT and GsdmD^−/−^ (KO) mice **a** at 6 h after hemorrhagic shock with resuscitation (HS/R), or **b** at 6 h after acetaminophen overdose (APAP). Quantitation of band density was performed across at least three separate blots and mean ± SEM is shown in the graphs (see below blots). **c** 4-HNE (lipid peroxidation) staining (red) in the liver from WT and GsdmD^−/−^ mice at 6 h after HS/R or APAP (blue = nuclei, green = actin). **d** Western blots of full-length caspase-8 (Full-cas8) and cleaved caspase-8 (Cleaved-cas8) in whole-cell lysates from WT hepatocytes at 1 h after hydrogen peroxide (H_2_O_2_) treatment at concentrations of 0.2, 1 or 5 mM. **e** Western blots of full-length caspase-8 (Full-cas8) and cleaved caspase-8 (Cleaved-cas8) in whole-cell lysates from WT hepatocytes at 1 h after 5 mM H_2_O_2_ treatment and *N*-acetyl cysteine (NAC) antioxidant given at concentrations of 0–5 mM. GAPDH used as loading control. Western blot images representative of results from at least three repeats. Each lane on Westerns contains liver from one individual mouse. Immunofluorescent images representative of multiple fields across mice from the same experimental groups. *N* = 4–5/group. *p < 0.05 between the indicated groups

Previous studies have reported caspase-8 as a key regulator of both apoptosis and necroptosis pathways^25,26^. Cleavage of caspase-8 favors induction of apoptotic cell death, while accumulation of uncleaved caspase-8 facilitates necroptotic cell death through direct interaction with the RIPK1/RIPK3 complex^26–28^. In other words, cleavage of pro-caspase-8 leads to increased cleaved caspase-8 with concomitant decreased pro-caspase-8 and induction of apoptosis, while high levels of pro-caspase-8 inhibit apoptosis and are associated with necroptosis. Recent studies have also demonstrated that caspase-8 is upregulated during infection-induced inflammasome activation in the absence of either caspase-1 or GsdmD^21^. Thus, to determine whether the differentially regulated cell death pathways in our models are under caspase-8 regulation, we measured expression and cleavage of caspase-8 in livers after HS/R or APAP overdose. Immunoblot analysis showed that, after 6 h HS/R, pro-caspase-8 undergoes cleavage in both WT and GsdmD^−/−^, generating the 43- and 17-kDa caspase-8 cleavage products (Fig. 4a). Interestingly, we found that GsdmD^−/−^ liver had increased cleaved caspase-8 compared with WT (Fig. 4a), consistent with higher levels of cleaved caspase-3 in GsdmD^−/−^ liver after 6 h HS/R, suggesting the higher liver damage in GsdmD^−/−^ mice after HS/R is due to increased activation of apoptosis. At 24 h after HS/R, however, both WT and GsdmD^−/−^ liver showed little or no cleaved caspase-8, in accordance with histological recovery of liver damage after about 24 h in HS/R, probably due to resolution of the hypoxic stimulus (Supplemental Fig. 1d). We still see increased apoptotic cells in GsdmD^−/−^ mice at later time points (Supplemental Fig. 1b), suggesting this increased damage is reflective of a larger initial injury.

Similarly, we measured liver caspase-8 after 6 h of APAP overdose. As opposed to HS/R, cleavage of pro-caspase-8 was reduced, resulting in accumulation of the pro-form of the enzyme. Only a relatively small portion of liver caspase-8 was cleaved in both WT and GsdmD^−/−^ mice compared with controls. GsdmD^−/−^ liver had significantly higher levels of both pro- and cleaved caspase-8 after APAP overdose (Fig. 4b) compared with WT after APAP, or WT or GsdmD^−/−^ liver after HS/R. We saw similar results at 12 h APAP overdose, likely as APAP continues to damage cells and continues to inhibit caspase-8 cleavage (Supplemental Fig. 1e). These data are in line with our findings of activation of necroptosis as the major cause of hepatocyte cell death in GsdmD^−/−^ mice after APAP overdose.

Together, these data show caspase-8 levels are increased in GsdmD^−/−^ liver during noninfectious injuries compared with WT, leading to increased liver damage in GsdmD^−/−^ mice. Cleavage levels of caspase-8 are modulated by model severity and this regulates levels of liver apoptosis or necroptosis.

We next asked what causes differential regulation of caspase-8 cleavage during HS/R and APAP overdose. Others have suggested the cysteine critical for proteolytic activation of caspases is susceptible to inhibition by either oxidation or nitrosylation^29^. As ROS are one of the major causes of liver damage during both HS/R and APAP overdose^8,30^, we asked whether oxidative stress level during noninfectious injuries affects caspase-8 cleavage, and subsequent apoptosis or necroptosis. Lipid peroxidation level, shown by 4-hydroxynonenal (4HNE), confirmed oxidative stress is significantly higher in APAP overdose compared with HS/R (Fig. 4c). To simulate different levels of oxidative stress, we treated cultured, isolated primary mouse hepatocytes with increasing doses of hydrogen peroxide (H_2_O_2_). Immunoblotting shows caspase-8 expression is elevated after H_2_O_2_ treatment, and cleavage of caspase-8 is inhibited by H_2_O_2_ in a dose-dependent manner. Higher doses of H_2_O_2_ resulted in reduced caspase-8 cleavage and increased pro-caspase-8 accumulation (Fig. 4d). To confirm, we used *N*-acetylcysteine (NAC) to reverse H_2_O_2_-induced oxidative stress, which also reversed caspase-8 protection from cleavage. Indeed, we found that NAC treatment rescued caspase-8 cleavage inhibition in a dose-dependent manner, with higher doses of NAC resulting in increased cleaved-caspase-8 after 1 h of 5 mM H_2_O_2_ (Fig. 4e).

**Fig. 5:**
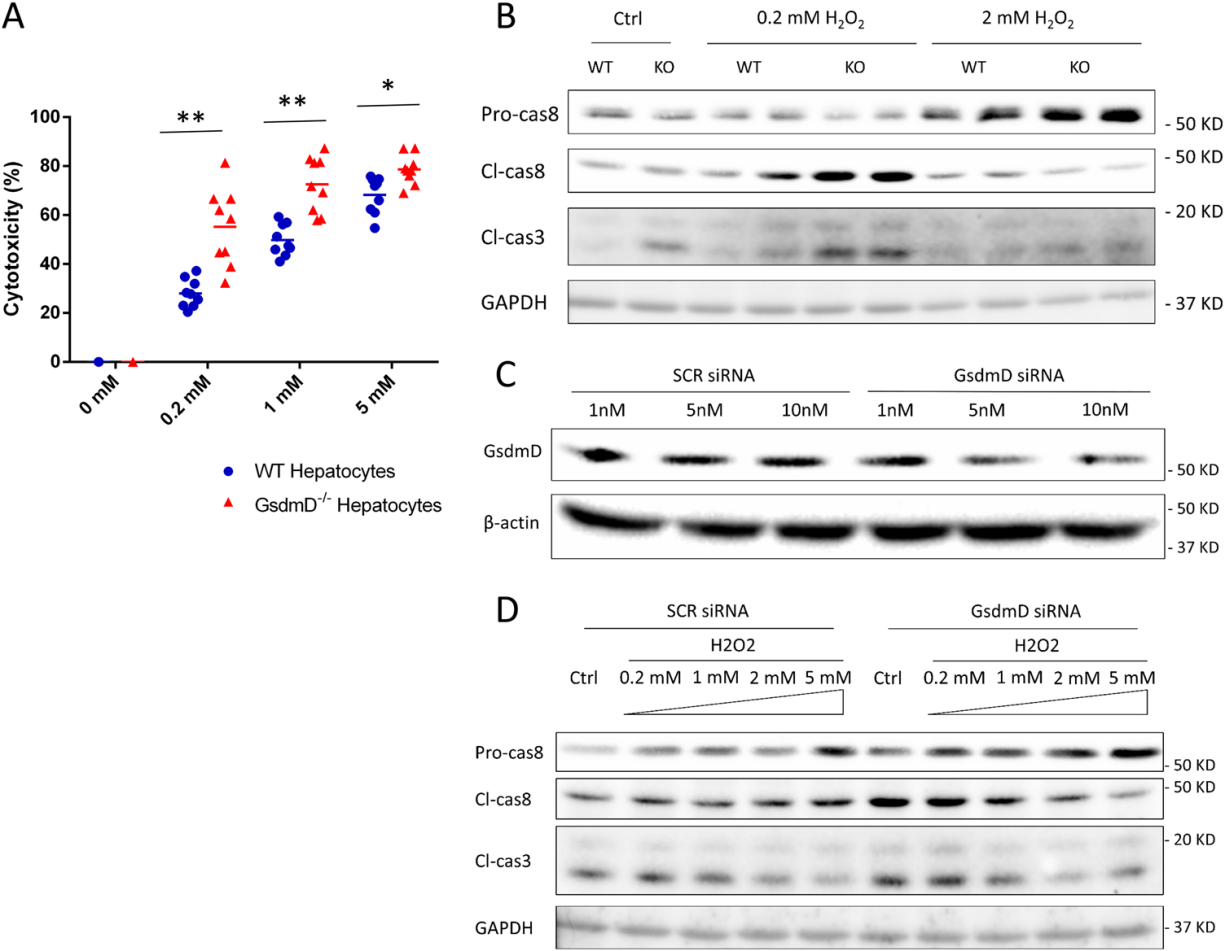
**a** LDH release (lytic cell death) in WT and GsdmD^−/−^ (KO) hepatocytes at 1 h after hydrogen peroxide (H_2_O_2_) at concentrations from 0–5 mM. Each data point represents levels from individual wells from at least three separate experiments and hepatocytes from at least three separate mice. **b** Western blots of full length (pro-)caspase-8 (Pro-cas8), cleaved caspase-8 (Cl-cas8) and cleaved caspase-3 (Cl-cas3) in whole cell lysates from WT and GsdmD^−/−^ (KO) hepatocytes at 1 h after low concentration (0.2 mM) or high concentration (5 mM) H_2_O_2_ or control PBS treatment (Ctrl). **c** Western blots of full length GsdmD in whole cell lysates from WT hepatocytes pretreated with control/scrambled siRNA (SCR siRNA) or GsdmD siRNA at concentrations between 1 and 10 nM. β-actin used as loading control. **d** Western blots of full length (pro-)caspase-8 (Pro-cas8), cleaved caspase-8 (Cl-cas8) and cleaved caspase-3 (Cl-cas3) in whole cell lysates from WT and GsdmD^−/−^ (KO) hepatocytes pretreated with control/scrambled siRNA (SCR siRNA) or GsdmD siRNA followed by 1 h of H_2_O_2_ at concentrations between 0.2 and 5 mM. GAPDH used as loading control. Images representative of results from at least three repeats. *p < 0.005 between indicated groups; **p < 0.001 between the indicated groups

To further test whether the hepatic protection mediated via GsdmD is through an intrinsic hepatocyte mechanism, we stimulated WT and GsdmD^−/−^ hepatocytes with concentrations of H_2_O_2_ and measured cytotoxicity (LDH release). Similar to in vivo, GsdmD^−/−^ hepatocytes showed significantly increased cytotoxicity during H_2_O_2_ treatment, suggesting that GsdmD protection is a hepatocellular intrinsic mechanism and loss of GsdmD renders hepatocytes more susceptible to oxidative stress (Fig. 5a). However, this does not rule out the possibility that the liver NPCs play a role in mediating the GsdmD-mediated hepatic protection during in vivo conditions. Immunoblotting also showed that at lower H_2_O_2_ concentration (0.2 mM), GsdmD^−/−^ hepatocytes had significantly increased caspase-8 cleavage, as well as increased cleaved-caspase-3 compared with WT, indicating upregulation of apoptotic cell death similarly to HS/R (Fig. 5b). At higher levels of H_2_O_2_ however, caspase-8 cleavage is inhibited and GsdmD^−/−^ hepatocytes had significantly increased accumulation of pro-caspase-8 compared with WT. To further validate regulation of caspase-8 by GsdmD, we used GsdmD siRNA to knock down expression of GsdmD in WT hepatocytes. A knockdown efficiency of about 50% was achieved at a concentration of 5 nM (Fig. 5c), which was the dose used in subsequent experiments. Similarly to GsdmD^−/−^ mice, GsdmD knockdown upregulated caspase-8, and caspase-8 cleavage was tightly regulated by levels of oxidative stress, confirming an alternative pathway of caspase-8 regulation by GsdmD.

To test whether the GsdmD regulates caspase-8 transcription, we measured *casp8* mRNA level. Interestingly, both the WT and GsdmD^−/−^ hepatocytes showed decreased *casp8* mRNA after H_2_O_2_ treatment with no significant difference between the two strains (Supplemental Fig. 2c). Similar results were observed in GsdmD-knockdown hepatocytes (Supplemental Fig. 2d). These data suggest that regulation of caspase-8 by GsdmD is not mediated at a transcriptional level and is more likely mediated through regulation of protein stabilization and degradation.

## Discussion

GsdmD was initially identified as a downstream substrate of caspase-11 during intracellular LPS sensing^6^. It is now known that GsdmD is essential for inducing pyroptosis by forming pores in cell membranes upon proteolytic activation and oligomerization of GsdmD N-terminal domain (GsdmD-N)^3^. Cell rupture causes release of cytokines, including IL-1β and IL-18, along with other damage-associated molecular patterns (DAMPs)^5^. GsdmD-N preferentially associates with phosphatidylserine (inner cell membrane) and cardiolipin (bacterial and mitochondrial membranes)^4^, so pore formation exhibits both cytotoxic and bactericidal functions during infectious injuries. GsdmD^−/−^ mice have improved mortality during lethal endotoxemia^6^, possibly due to reduced pro-inflammatory content release via pyroptosis. In intracellular bacterial infections, however, mutation in GsdmD protein resulted in compromised bacterial clearance during *L.monocytogenes* infection^4^.

To determine the role of GsdmD in noninfectious injuries, we deployed two clinically relevant sterile liver inflammation models with different severities: HS/R, simulating traumatic blood loss and causing a mild liver injury, which resolves over time, and APAP-induced liver injury, causing massive and longer-lasting liver damage. In contrast to the detrimental effect of GsdmD during lethal endotoxemia, our data indicate that GsdmD has a hepatoprotective effect during noninfectious injuries. Higher pro-inflammatory cytokine IL-6 and HMGB-1 levels in GsdmD^−/−^ mice suggest exacerbated liver damage and associated increased secondary inflammation in the absence of GsdmD.

Multiple cell death pathways including apoptosis, pyroptosis and necroptosis have been shown to mediate liver damage during noninfectious liver injuries^17,31,32^. Therefore, we investigated whether the absence of pyroptosis will result in a compensatory upregulation of other forms of cell death. Our data revealed a protective effect of GsdmD involving inhibition of both apoptosis and necroptosis, and dependent on the severity of the injury inducing the liver damage. The current study did not investigate the complex interplay of apoptosis and necroptosis within individual cells, but this is obviously an important and interesting concept. Future studies will be important to determine how the two pathways, and potentially others, are activated in a dynamic way within each cell, and how this determines the actual mode of cell death in an injury-dependent manner.

Caspase-8 is a key regulator of multiple cell death pathways. It mediates apoptosis upon cleavage and activation, but inhibition of proteolytic activation of caspase-8 leads to phosphorylation of RIPK1/RIPK3 complex and the necroptosis^26,28^. Recent studies revealed caspase-8 upregulation during infections in the absence of either caspase-1 or GsdmD^21^. Our data show that GsdmD^−/−^ mice also have increased caspase-8 expression after noninfectious injuries, with different regulation of caspase-8 cleavage dependent on ROS level and severity of injury, which ultimately determines the main type of cell death pathway. Pro-caspase-8 is readily cleaved after HS/R (minor injury), so facilitating increased apoptosis. However, after APAP overdose caspase-8 cleavage is largely inhibited, resulting in accumulation of pro-caspase-8 with some caspase-8 cleavage leading to increased necroptosis and apoptosis. Necroptosis seems to be the major cell death pathway in more severe oxidative stress injury, and results in more severe liver damage.

Previous studies suggested oxidation of the cysteine critical for proteolytic activation of caspases reduces its activation/cleavage^33^. Similarly, absence of antioxidant enzyme glutathione peroxidase 4 (Gpx4) inactivates caspase-8, resulting in necroptosis in hematopoietic cells^28^. Our hepatocyte in vitro results are consistent with these findings, with caspase-8 cleavage inhibited by H_2_O_2_ in a dose-dependent manner, and NAC able to reverse caspase-8 cleavage inhibition also in a dose-dependent manner. Taken together our data suggest pivotal redox regulation of cell death pathways through caspase-8, which may be modulated by GsdmD. Not only does this provide additional mechanistic interpretation of NAC protection in APAP overdose, but it also opens the possibility of development of additional therapeutics for APAP overdose targeting caspase-8 or GsdmD.

Caspase-8 can also play an important role in inflammasome formation^34^, and it is proposed that caspase-8 is able to cleave GsdmD to induce pyroptosis in certain conditions such as Yersinia infection^35,36^. Thus, we speculate that the deficiency of GsdmD might affect caspase-8 expression through an intrinsic cellular feedback regulation. Other studies have also suggested that caspase-8 expression can be induced by cytokines including IFNγ,^37^ thus sensitizing the cell for caspase-8-dependent cell death. Therefore, it is also possible that GsdmD deficiency can affect liver caspase-8 level through in a cytokine-dependent manner.

In summary, our study revealed an unappreciated hepatoprotective role of GsdmD during noninfectious liver injuries through downregulation of caspase-8-induced apoptosis and necroptosis. The protection exists in different noninfectious injury models with different severities, yet the specific mechanism varies and is model specific. Our study also provides new insights into fine-tuning of different cell death pathways through caspase-8 cleavage in liver, although the specific regulation of capspase-8 by GsdmD requires further investigation. Pharmacological manipulation of the GsdmD/caspase-8 axis could be beneficial in clinical courses of noninfectious liver injuries.

## Supplementary information

Supplemental Figure Legends

Supplemental Figure 1

Supplemental Figure 2
