# Supplementary material for: Gasdermin D protects against noninfectious liver injury by regulating apoptosis and necroptosis
Source: Cell Death Dis. 2019 Jun 17;10(7):481. doi: 10.1038/s41419-019-1719-6 (PMC6579760; doi:10.1038/s41419-019-1719-6)
Supplement: Supplementary file 1 — Supplemental Figure Legends [file 41419_2019_1719_MOESM1_ESM.docx]

**Supplemental Figure Legends**

**Supplemental Figure 1:** **(A)** Liver histology (H&E) in WT and GsdmD^-/-^ (KO) liver at 24h after hemorrhagic shock with resuscitation (HS/R) or 12h after acetaminophen overdose (APAP). **(B)** Liver immunofluorescent TUNEL staining in WT and GsdmD^-/-^ liver at 24h after HS/R or 12h after APAP. Red=TUNEL positive cells; Blue= nuclei; Green=actin. Scale bar, 40 μm. Magnification = 20x. Dotted lines in H&E images outline necrotic areas. All images representative of each group. N=3/group. **(C)** Quantification of liver histology and TUNEL staining (1.38±0.56 vs 4.30±0.88; WT vs GsdmD^-/-^; n=3, p=0.0491) (13.85±6.63 vs 51.32±4.88; WT vs GsdmD^-/-^; n=3, p=0.0104) (0.51±0.33 vs 4.69±0.60; WT vs GsdmD^-/-^; n=3, p=0.0035) (10.01±0.64 vs 13.31±4.46; WT vs GsdmD^-/-^; n=3, p=0.5042). Western blots of full length (pro-)caspase-8 (Pro-cas8) and cleaved caspase-8 (Cl-cas8) in whole cell lysates from liver of WT and GsdmD^-/-^ (KO) mice **(D)** at 24h after hemorrhagic shock with resuscitation (HS/R), or **(E)** at 12h after acetaminophen overdose (APAP) and western blots of total RIPK1 (RIPK1) and phosphorylated RIPK1 (p-RIPK1) as markers of necroptosis.

**Supplemental Figure 2:** Western blots of whole cell lysates from liver of WT and GsdmD^-/-^ (KO) mice at 24h after hemorrhagic shock with resuscitation (HS/R) or sham surgery (Sham), or 12h after acetaminophen overdose (APAP) or controls. (A and B) full length (pro-)caspase-3 (Pro-cas3) and cleaved/active caspase-3 (Cl-cas3) as a marker of apoptosis. (C and D) qPCR of CASP8 gene relative expression in whole cell lysates of hepatocytes at 1h after 2mM H_2_O_2_ or control PBS treatment (Ctrl) and after pretreated with control/scrambled siRNA (SCR siRNA) or GsdmD siRNA followed by 1h of H_2_O_2_ at concentration of 2mM. NS=not significant
