# Supplementary figures and images for: Gasdermin D protects against noninfectious liver injury by regulating apoptosis and necroptosis
Source: Cell Death Dis. 2019 Jun 17;10(7):481. doi: 10.1038/s41419-019-1719-6 (PMC6579760; doi:10.1038/s41419-019-1719-6)

# Supplement Figure 1

A

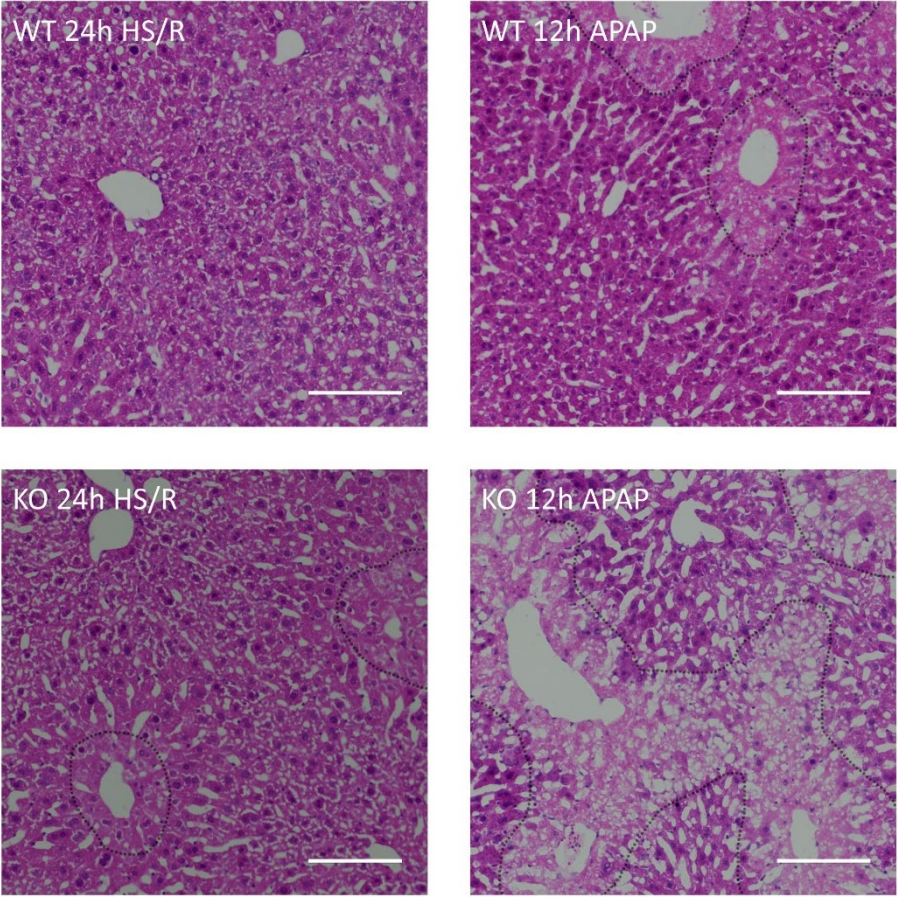

B

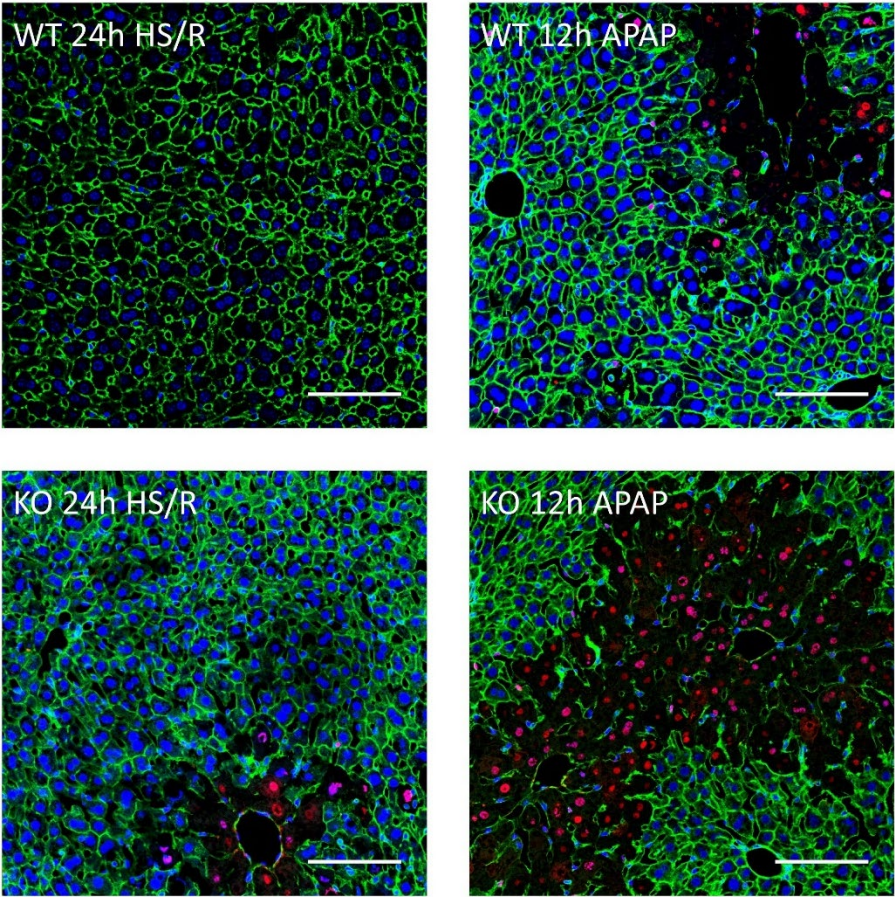

C

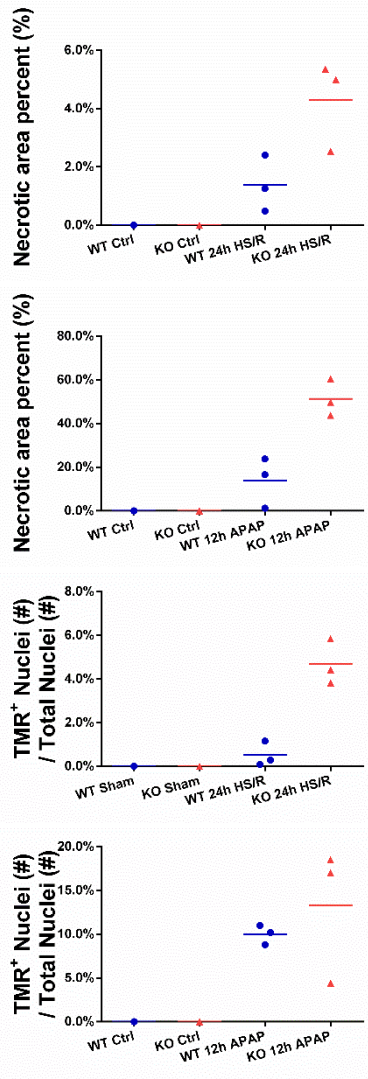

D

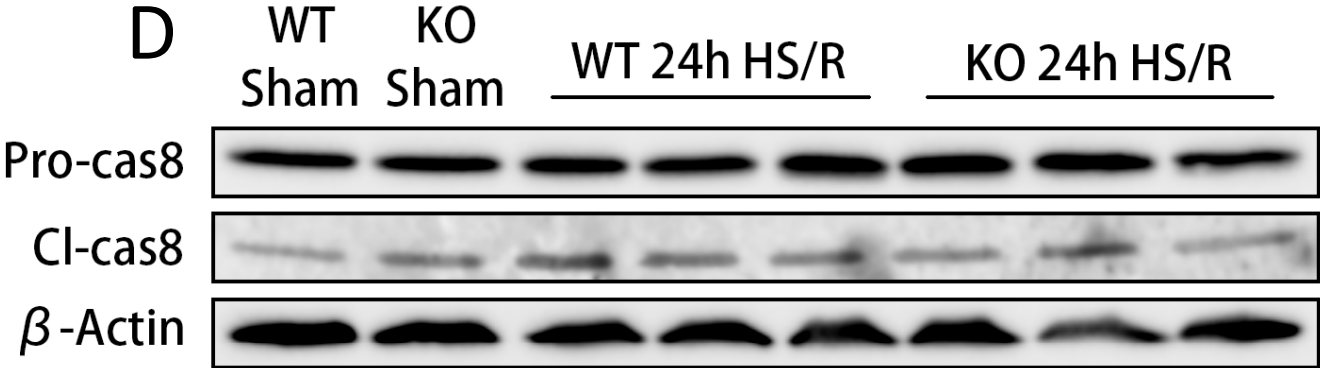

E

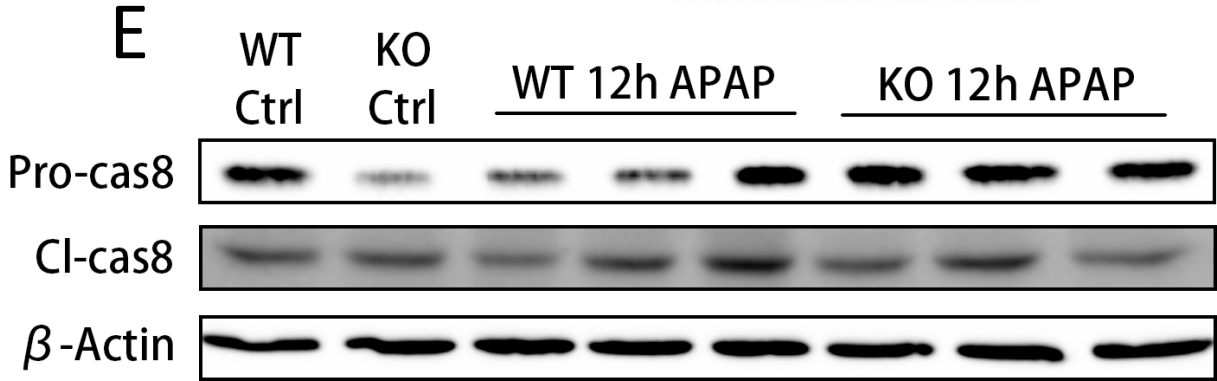

F

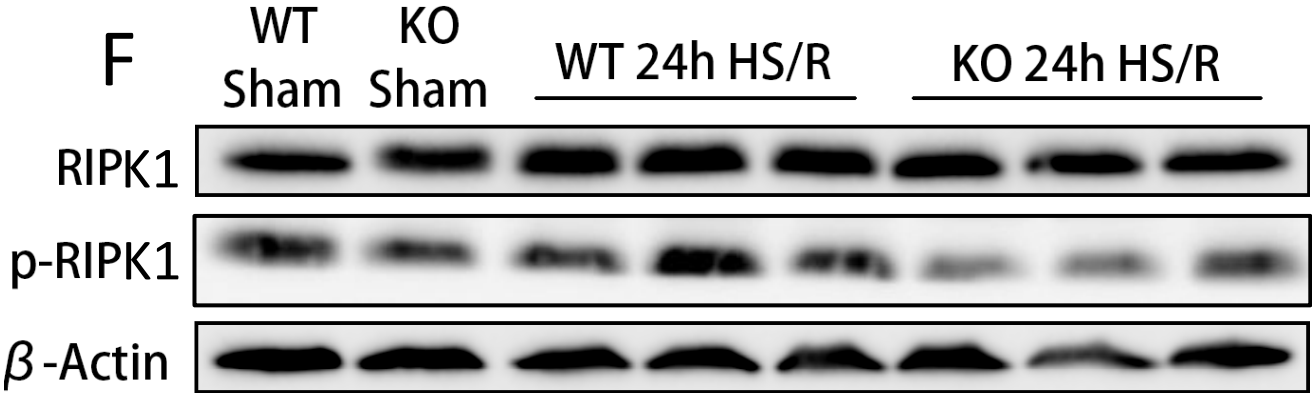

G

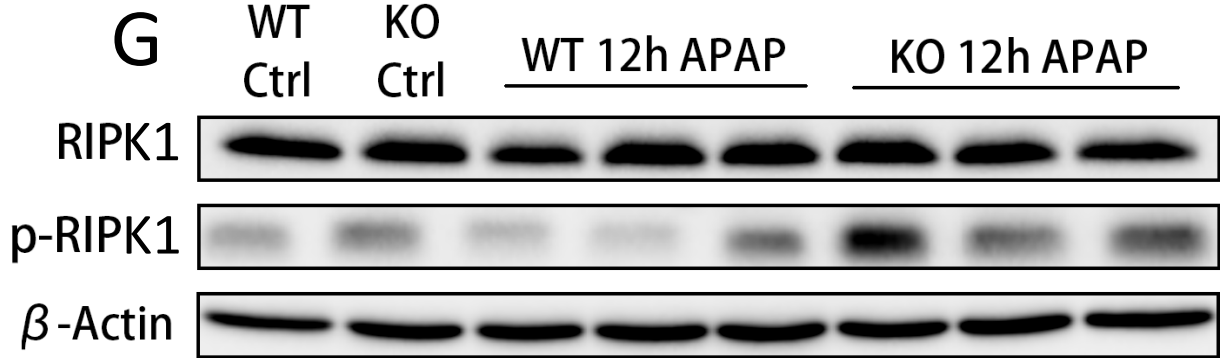

Supplement: Supplementary file 2 — Supplemental Figure 1 [file 41419_2019_1719_MOESM2_ESM.pdf]

Supplement Figure 2

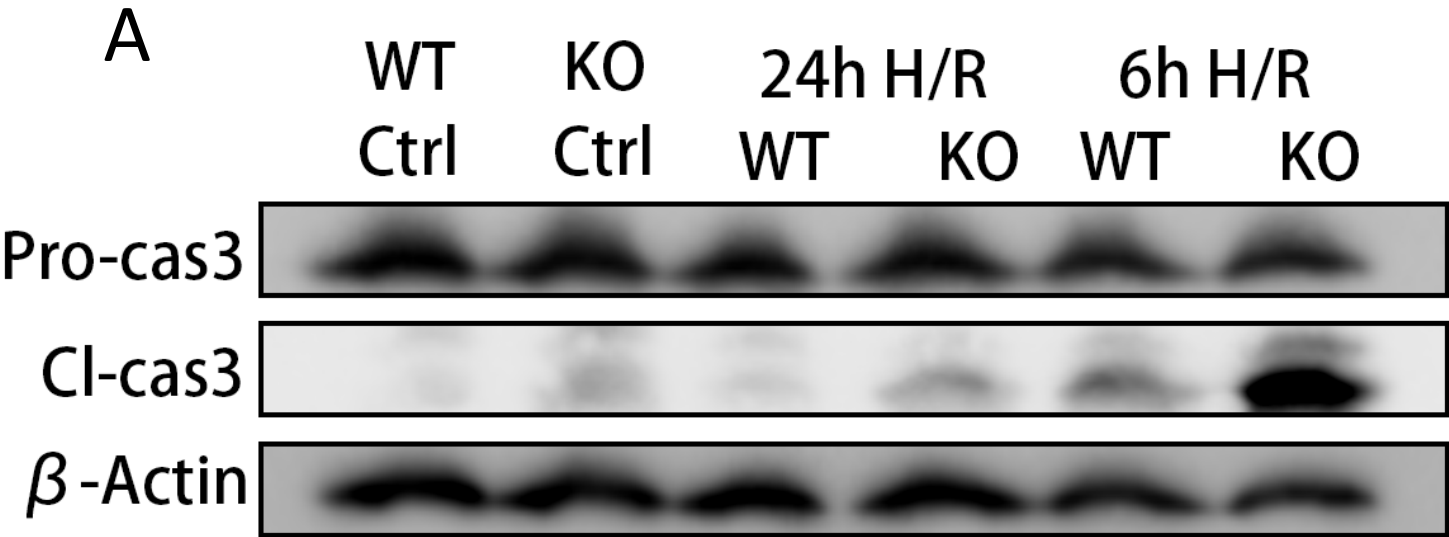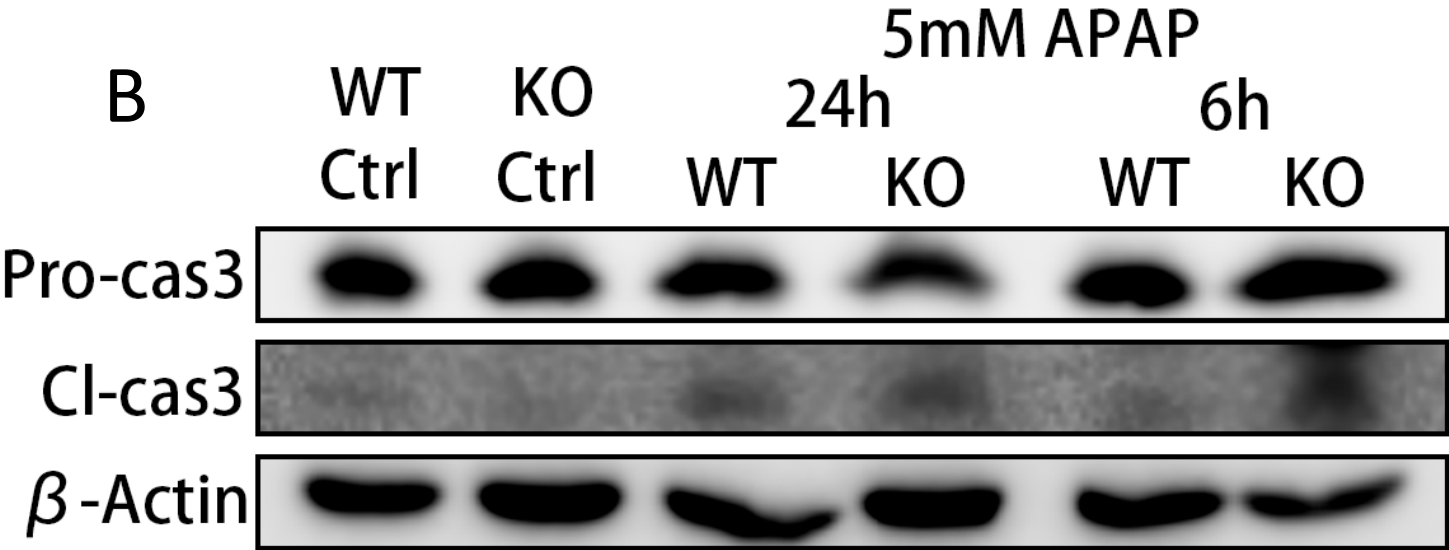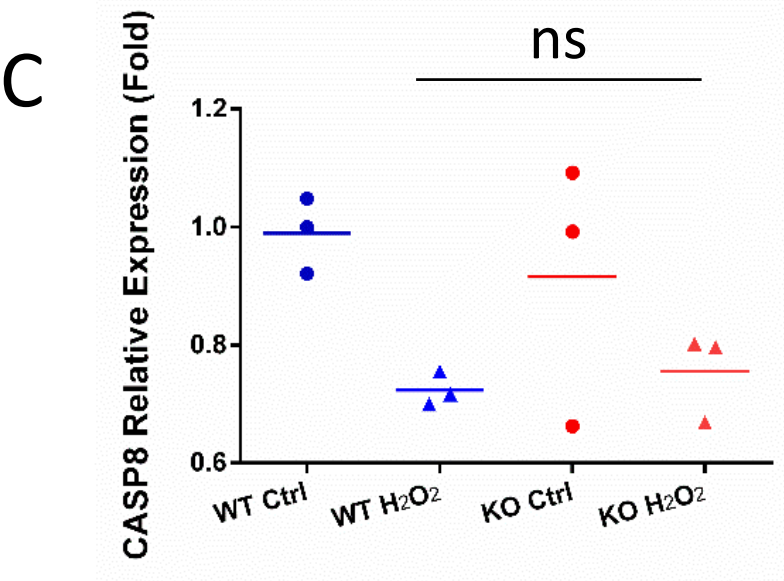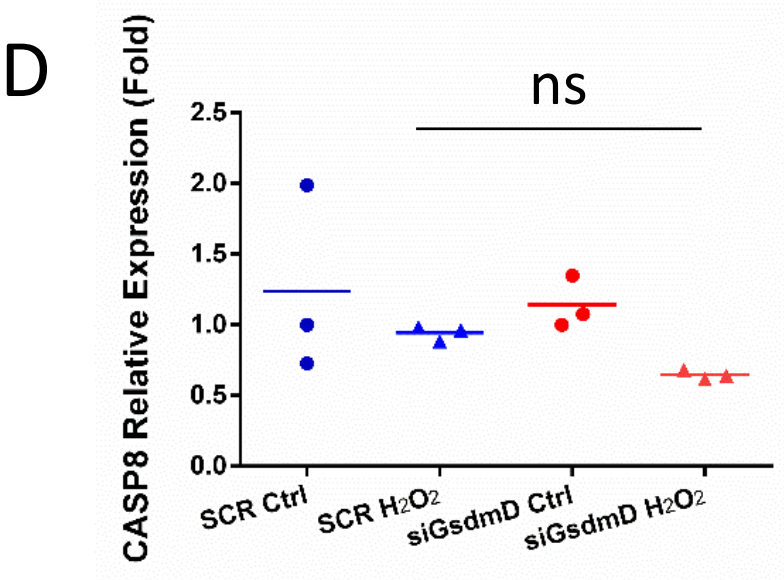

Supplement: Supplementary file 3 — Supplemental Figure 2 [file 41419_2019_1719_MOESM3_ESM.pdf]
